# Abnormal Large Central Occipital Emissary Vein: A Case Report and Review of Literature

**DOI:** 10.7759/cureus.603

**Published:** 2016-05-08

**Authors:** Mohamed Salem, Parviz Dolati, Matthew R Fusco, Christopher S Ogilvy, Ajith J. Thomas

**Affiliations:** 1 Neurosurgery, BIDMC Harvard Medical School; 2 Vanderbilt University Medical Center

**Keywords:** occipital, emissary, vein, anatomic variation, torcula

## Abstract

A detailed description of the anatomy of the central occipital emissary vein, its embryology, anatomy, and abnormal variations is not available in the literature. This is the first known case report. A 48-year-old female underwent cerebral angiography to rule out dural arterio-venous fistula. Her angiography revealed an abnormally large central occipital emissary vein originating from the torcula, penetrating the cranium and draining into the suboccipital venous plexus. We provide discussion of the case with a review of the related literature. This case and its attached radiological images introduce a new type of entity to the existing data about the cranial emissary veins.

## Introduction

Emissary veins are surgically important structures that receive surprisingly little attention in the medical literature. These veins can provide surgical obstacles that cause excessive bleeding if not properly prepared for, particularly in pediatric posterior fossa cases. In addition, as demonstrated in this case, emissary veins can often be mistaken for outflows in dural AV fistulae when in reality they are simply anatomic variants. Anomalies of three main posterior fossa emissary veins have been reported: mastoid, parietal, and petro-squamous emissary veins. We report a rare anomaly of a midline occipital emissary vein with review of previous similar reports. Informed consent was obtained from the patient for this study.

## Case presentation

**Figure 1:**
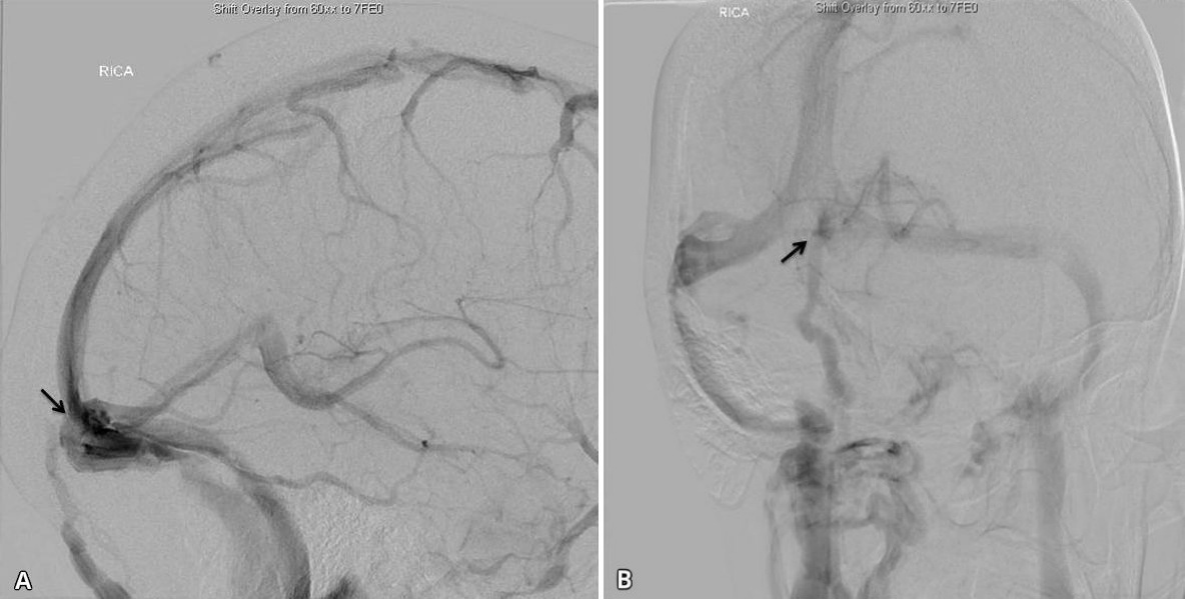
Lateral (A), Anteroposterior (B) views of the right ICA injection demonstrate the central occipital emissary vein (arrows) originating from the torcula & draining into the suboccipital venous plexus.

**Figure 2:**
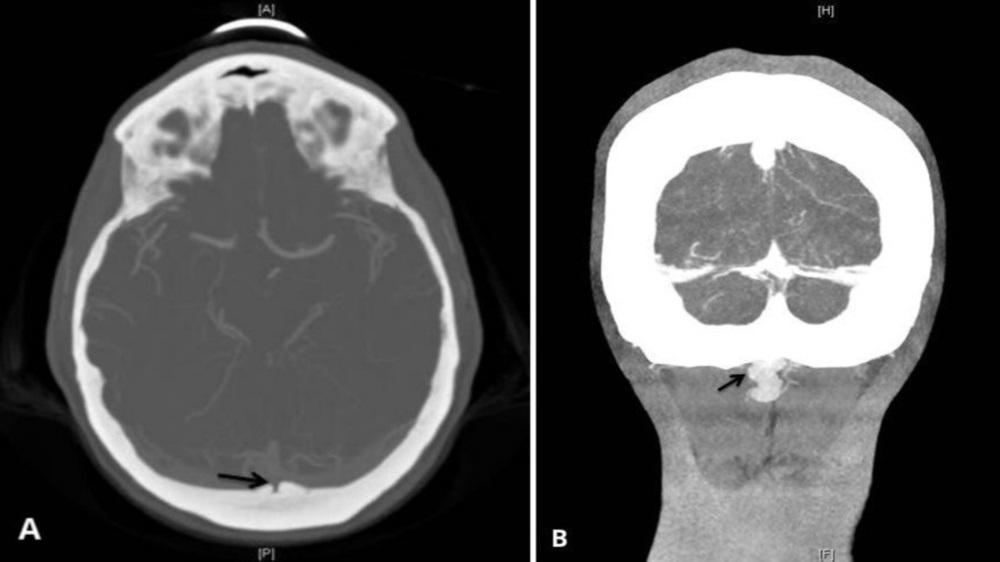
Axial (A), Coronal (B) views of CTA showing the penetration site of the central occipital emissary vein (arrows) to the cranium.

This patient is a 48-year-old female who had been complaining of achiness involving the left upper extremity and pins and needles in the left palm for eight months. She also noted intermittent blurring of her vision. A magnetic resonance imaging (MRI) of the C-spine showed multiple flow voids and prominent arteries in the occipital and suboccipital regions. These imaging findings prompted referral for a neurosurgical consultation. Neurologic examination was normal except deep tendon reflexes which were exaggerated. Given her young age, upper limb symptoms and hyperreflexia, an angiogram was done to rule out a dural AV fistula. The right internal carotid artery angiogram clearly demonstrated a transosseous vein draining from the torcula through the occipital bone into the suboccipital cranial plexus with no drainage into the coronal venous plexus and no retrograde venous drainage into the spinal cord (Figure 1). This venous drainage from the torcula occurred during the normally timed venous phase. Multiple dilated venous channels were also noted in the suboccipital region (Figure 2). No further treatment was indicated as this finding did not have any bearing on the patient’s clinical presentation.

## Discussion

To our knowledge, this is the first case of an extremely large and tortuous midline occipital emissary vein (EV) from the torcula reported in the English-language medical literature. A dural arterio-venous fistula (DAVF) can easily be mistaken with this type of EV. Having enough embryological and anatomical knowledge about these normal variants of EV is absolutely necessary for all neurosurgeons and neurointerventionists.

The EVs are valveless structures that connect the intracranial dural sinuses with extracranial veins and can often demonstrate important clinical implications. The embryological mechanism for formation of the emissary veins is related to the primary capillary plexus of the head which becomes separated into three fairly distinct strata by the differentiation of the skull and meninges. The superficial vessels, draining the skin and underlying soft tissue, eventually drain in large part into the external jugular system. Dural sinuses arise from the middle layer, with the deepest layer forming the cerebral veins. Emissary veins are formed through retained residual connections between the superficial and middle layers [1-2].

The majority of cerebral venous drainage in humans is through internal jugular veins and vertebral venous plexuses, with a limited role for the external jugular vein [3]. Postural differences in cerebral venous drainage have been reported, with the internal jugular veins being more dominant in the supine position. While in the erect position, the internal and external vertebral venous system constitutes the primary venous pathway. Emissary veins play a role in connecting the dural sinuses with the vertebral system [4]. Three kinds of posterior fossa emissary veins have been described in the literature: mastoid emissary vein (MEV), posterior condylar vein, petrosquamosal sinus (PSS). Information regarding midline occipital emissary vein (OEV) is lacking in the literature.

The role of emissary veins in non-pathologic conditions often goes unnoticed clinically. On the contrary, in cases of abnormal pathologic conditions the emissary veins can provide vital function. Selective brain cooling by emissary veins has been reported as a method to cool the venous blood in the brain as a protection from acute thermal damage [5]. In cases of skull based masses, vascular malformations, or jugular vein outflow obstruction, emissary veins can provide a safety valve for cerebellar venous outflow to prevent an increase in intracranial pressure. One report demonstrated five out of six patients with an enlarged PSS to have associated jugular vein hypoplasia [2].

Although our patient did not present with tinnitus, this can be a presenting complaint of an enlarged EV [6]. Chauhan et al. [7] reported a 30-year-old female presenting with tinnitus. Workup of the patient revealed persistent emissary veins including a dilated left MEV, bilateral PSS, posterior condylar veins, and OEV. Abnormal origin of a left PSS from the MEV was also described, along with posterior fossa sinuses anomalies.

EVs may serve as a pathway for intracranial transmission of infection. Mortazavi et al. [8] reviewed multiple ways in which emissary veins can be involved in spreading the infection to the intracranial cavity. The implication of the parietal emissary vein in the "danger area of scalp" was highlighted in their review. This area is located in the loose areolar connective tissue layer of the scalp. EV opening into this layer is a potential pathway for spreading of different infectious agents into the intracranial dural sinuses. The spreading of infection or metastasis from the auditory canal via the petrosquamosal sinus has been described [8]. Moreover, communication of the orbital EV with the ophthalmic vein is a well-described route for cavernous sinus infection and subsequent thrombosis [8].

One of the interesting hypotheses regarding the mechanism of dural AV fistula formation implicated the role of emissary veins in the formation of the fistula [9]. Per this hypothesis, local inflammation at the penetration site of the EV to the dura creates neovascularization and AV shunt formation at the arteriolar level. The increase in shunted flow triggers drainage into the sinus. Subsequent degradation of the emissary vein due to compression by enlarged arteries results in an impairment of blood flow. Angiogenic factors are then expressed, leading to expansion of the shunt to the surrounding dura and recruitment of distant feeders that ultimately perpetuate the hemodynamic imbalance [9].

From the surgical point of view, enlarged EVs are an important source of bleeding in cranial procedures if missed preoperatively. Ahmad et al. [10] reported a case of missed giant MEV which was opened while operating on the mastoid area and caused profuse hemorrhage which was controlled only by surgical packing. Other surgical implications of EVs were mentioned in the review by Mortazavi et al. [8]; they discussed the importance of EVs in transcondylar and retrosigmoid approaches. Therefore, the emissary veins should be freed from the bone to avoid avulsion or laceration of the sinuses which can lead to thrombosis or air embolism. Emissary veins are also important in endovascular procedures; Rivet et al. [11] described a technique of using the mastoid EV for embolization of a Borden-Shucart type II dural arteriovenous fistula (DAVF) in the transverse sigmoid sinus junction. However, the interventionist should be cognizant of large EV variants and not treat these as a DAVF.

**Figure 3:**
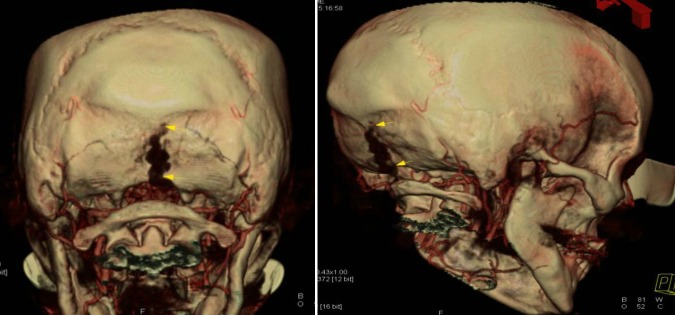
3D reconstruction of the CTA showing the occipital emissary vein course to the suboccipital venous plexus.

The reported case here demonstrates many of the important aspects of posterior fossa emissary veins (Figure 3). Although initial outside hospital reports claimed the enlarged extracranial veins represented a DAVF, catheter angiography clarified the anatomy and direction of venous flow and ruled out an abnormal AV fistula. Otherwise, had surgical exploration been pursued, significant difficulty to control venous bleeding would have been encountered. In addition, disruption of normal cranial venous egress would have occurred. 

## Conclusions

Cranial emissary veins are normal venous structures that have critical surgical importance and those occurring within the posterior fossa should be carefully examined before occipital, suboccipital, and mastoid surgeries to prevent unexpected bleeding. Emissary vein variations can create significant surgical complications if not properly identified or be mistaken for AV fistulae. Increased knowledge of their embryology, anatomy, abnormal variations, and clinical implications is essential for all neurosurgeons and neurointeventionists.
